# Supplementary material for: Alcohol Misuse: Integrating Personality Traits and Decision-Making Styles for Profiling
Source: Behav Sci (Basel). 2025 May 2;15(5):622. doi: 10.3390/bs15050622 (PMC12108872; doi:10.3390/bs15050622)
Supplement: Supplementary file 1 [file behavsci-15-00622-s001.zip › behavsci-3452531-supplementary.pdf]

Supplemental material

Table S1. Correlations of Age, Sex, alcohol misuse (AUDIT and RAPI), personality scales (ZKA-PQ/SF, BIS-11, UPPS, and NEO-PI-R), and decision-making scales

	1	2	3	4	5	6	7	8	9	10	11	12	13	14	15	16	17	18	19	20	21	22	23	24	25
1. Age	1																								
2. Sex	-.02	1																							
3. Audit	-.29	-.17	1																						
4. Rapi	-.31	-.09	**.67**	1																					
5. Aggressiveness	-.25	-.10	.25	.28	1																				
6. Activity	.10	.01	-.09	-.02	.02	1																			
7. Extraversion	-.06	.04	.01	-.06	-.15	.16	1																		
8. Neuroticism	-.21	.19	.10	.20	.38	.02	-.32	1																	
9. Sensation Seeking	**-.40**	-.16	.32	.31	.25	.18	.24	-.01	1																
10. Impulsivity (BIS-11)	-.31	-.06	.30	.32	**.43**	-.01	.05	.27	**.49**	1															
11. Negative Urgency	-.16	-.02	.16	.24	**.55**	.03	-.10	.35	.17	.39	1														
12. Lack of premeditation	-.16	-.03	.18	.19	.21	-.12	.04	.11	.28	**.54**	.29	1													
13. Lack of perseverance	-.25	-.05	.13	.19	.20	-.24	-.09	.26	.20	**.44**	.27	**.56**	1												
14. Sensation Seeking	-.39	-.15	.24	.28	.22	.11	.15	-.02	**.68**	.38	.24	.27	.21	1											
15. Positive Urgency	-.22	-.05	.27	.30	.37	.05	.04	.23	.34	**.44**	.51	.33	.28	.42	1										
16. Neuroticism	-.31	.16	.16	.24	**.51**	-.13	-.35	**.83**	-.02	.29	.40	.14	.29	.04	.29	1									
17. Extroversion	-.19	-.04	.15	.13	-.07	.31	**.76**	-.29	.47	.16	-.04	.08	-.10	.36	.13	-.28	1								
18. Openness	-.38	.09	.14	.14	-.03	.01	.25	.06	.42	.16	-.03	.08	.09	.31	.09	.09	.38	1							
19. Agreeableness	.31	.23	-.21	-.26	**-.54**	.03	.23	-.07	-.21	-.27	-.32	-.19	-.19	-.23	-.26	-.18	.06	-.02	1						
20. Conscientiousness	.27	.08	-.29	-.26	-.33	.39	.15	-.31	-.25	**-.66**	-.31	**-.56**	**-.57**	-.19	-.35	**-.42**	.13	-.05	.28	1					
21. Rational	.03	.06	-.12	-.17	-.22	.21	.08	-.09	-.16	**-.49**	-.28	**-.56**	-.34	-.15	-.27	-.19	.06	.11	.13	**.57**	1				
22. Intuitive	.08	.03	-.03	-.05	-.06	.14	.23	-.16	.10	.00	-.07	-.02	-.16	.00	-.03	-.18	.17	-.02	.09	.15	.10	1			
23. Dependent	-.07	.12	-.01	-.01	.04	-.01	.02	.34	-.09	.01	.11	-.02	.03	-.12	.07	.33	-.09	-.07	.23	-.08	.07	.05	1		
24. Avoidant	-.12	.04	.14	.20	.23	-.12	-.19	.42	.09	.37	.25	.26	.36	.05	.25	.43	-.17	-.01	-.12	**-.45**	-.27	-.14	.26	1	
25. Spontaneous	-.17	-.07	.21	.24	.30	.09	.03	.11	.40	.55	.28	.43	.26	.33	.31	.17	.13	.09	-.23	**-.44**	-.40	.12	-.06	.26	1

Correlations larger than +/- .40 are in boldface. Sex was coded as follows: 1 (male) and 2 (female). Note that the correlations with NEO-PI-R were computed for the specific subsample only
